# Tacrolimus-Associated Maculopathy in a Patient Following Kidney Transplantation

**DOI:** 10.7759/cureus.70157

**Published:** 2024-09-25

**Authors:** Sultan Almdallaleh, Abdulhadi Altalhi, Kheira Azzaz, Oudeh Oudeh, Mohammed Radwan

**Affiliations:** 1 Nephrology, King Saud Medical City, Riyadh, SAU; 2 Pediatric Nephrology, King Saud Medical City, Riyadh, SAU; 3 Ophthalmology, Cairo University, Cairo, EGY; 4 Ophthalmology, King Saud Medical City, Riyadh, SAU

**Keywords:** immunosuppressive therapies, ischemic maculopathy, kidney transplantation, sudden vision loss, tacrolimus

## Abstract

Tacrolimus is one of the mainstay medications for renal transplantation patients. Vision loss is a rare but serious adverse effect associated with tacrolimus use.

Herein, we report a male patient who developed acute vision loss during hospitalization for slow kidney graft function post-renal transplantation and 19 days after starting tacrolimus. The vision loss was associated with a recent rise in tacrolimus levels that required dose adjustment. The workup excluded cortical blindness and showed evidence of a newly developed bilateral central white cloudy inner retinal opacification resembling a cherry red spot on fundus examination. Tacrolimus toxicity was suspected, and the medication was replaced with cyclosporin. Follow-up was remarkable for mild improvement in the patient’s vision and disappearance of structural changes on fundoscopy in both eyes. These findings are in keeping with a diagnosis of ischemic maculopathy.

This is the first case of bilateral severe early-onset tacrolimus-associated ischemic maculopathy. According to our workup, this disease can be explained by a vasoocclusive accident at the arteriolar and venular level of the ciliomacular vascular system.

## Introduction

Tacrolimus is a calcineurin inhibitor (CNI) that inhibits the production of interleukin-2 and other cytokines in T lymphocytes [1], which is used as the mainstay immunosuppressant agent after kidney transplantation according to the Kidney Disease Improving Global Outcomes guidelines [2].

Vision loss is a rare but serious adverse effect of tacrolimus. There have been previous reports of posterior reversible encephalopathy syndrome (PRES), optic neuropathy, and maculopathy associated with tacrolimus use [3-6]. Herein, we present a case of blindness one month post renal transplant and 19 days after starting tacrolimus, with further workup suggestive of ischemic maculopathy. Only two cases of maculopathy associated with tacrolimus use were reported in the literature so far [4,5]. The patient provided informed consent for using his medical records for research purposes.

## Case presentation

A 43-year-old Saudi male presented to our hospital four days after living non-related kidney transplantation. He has had hypertension for more than 10 years and chronic kidney disease that has required renal replacement therapy since July 2022. He was on the following medications: cyclosporine 150 mg oral capsule twice daily, mycophenolate mofetil 1000 mg oral capsule twice daily, prednisolone 60 mg oral tablet once daily, and perindopril 10 mg oral tablet once daily. Upon arrival at the emergency department, the patient was clinically stable. Physical examination revealed a permanent right-sided internal jugular hemodialysis catheter and a clean surgical wound at the right iliac fossa without signs of infection. The abdomen was soft with a palpable, non-tender kidney graft in the right iliac fossa. The notable laboratory findings included an elevated serum creatinine (156 mg/dl), a maintained target cyclosporin level (C0: 155 ng/mL), pyuria (white blood cells: 5-10), and hematuria (red blood cells: 50-100). The patient was admitted for post-transplant care and evaluation of renal impairment. Table 1 summarizes the patient's laboratory investigations on the day of admission.

**Table 1:** Laboratory results on admission. LDH: lactate dehydrogenase; INR: international normalized ratio

	Result	Unit	Normal range
Serum creatinine	156	µmol/L	62-106
Blood urea nitrogen	15	mmol/L	2.50-7.01
Sodium	135	mmol/L	135-145
Potassium	4.85	mmol/L	3.5-5.1
Glucose	7	mmol/L	3.5-5.6
LDH	259	U/L	125-220
Albumin	35	g/L	35-52
Calcium	2	mmol/L	2.18-2.60
Phosphorus	0.9	mmol/L	0.80-1.45
White blood cells	8	10*3/uL	4-10
Hemoglobin	10	g/dL	12.2-18.2
Platelets	126	10*3/uL	150-410
Prothrombin time	15	sec	12.3-15.7
Partial thromboplastin time	21	sec	26-40
INR	1		
Cyclosporine level	155	ng/ml	100-400
Vitamin B12	411	pmol/L	138-652
Folic acid	6.58	nmol/L	7-46.4
Thyroid-stimulating hormone	0.63	mIU/L	0.53-4.94
Free thyroxine (FT4)	18.26	pmol/L	9.01-19.05
Vitamin D	44.53	nmol/L	Normal level: 75 -125; insufficiency: 51-74.9; deficiency: 0-50.9

During admission, cyclosporine was changed to immediate-release tacrolimus (total daily dose: 7 mg), and Bactrim and valganciclovir were started at prophylactic doses. Tacrolimus levels increased and reached 22 mcg/mL, and the dose was titrated to target levels of 8-12 mcg/mL. Virology screening for hepatitis B and C viruses, human immunodeficiency virus, cytomegalovirus, and polyomavirus BK were all negative. 

A kidney biopsy was planned to evaluate the slow graft function, but this was delayed because the patient developed a fever. Piperacillin-tazobactam was started empirically, then was changed later to vancomycin based on a blood culture report of methicillin-resistant Staphylococcus epidermitis. The internal jugular dialysis catheter was removed. Clinical improvement was seen afterward.

During the admission, 28 days after the kidney transplant, and 19 days after starting tacrolimus, the patient developed sudden bilateral painless visual loss that was not associated with neurological symptoms. Visual acuity was severely reduced bilaterally (counting fingers at 1 meter in the right eye, 20/40 in the left eye). Anterior segment examination on slit lamp was normal bilaterally, with normal eyelids, clear cornea, and depth of anterior chamber. The iris had a normal color and pattern, and the pupil examination was normal bilaterally. The lenses were in place, and the intraocular pressure was normal bilaterally. The fundus was unexpectedly normal with a clear vitreous, flat retina, normal retinal vasculature, and normal optic nerve. The vision loss was suspected to have a cortical etiology, but brain magnetic resonance imaging (MRI) showed no abnormal findings to support this.

**Figure 1:**
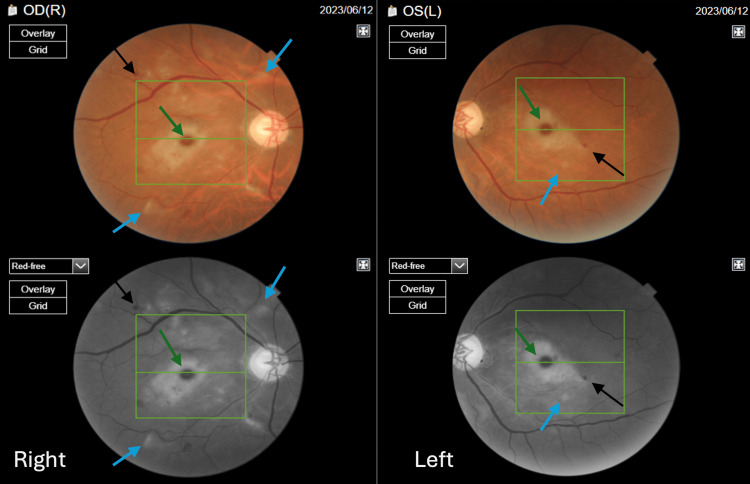
Color and red-free fundus photography of both eyes four days after symptom onset. Bilaterally, a central white cloudy inner retinal opacification resembling a cherry red spot is seen (green arrows) alongside central cotton wool spots (blue arrows) and blot hemorrhages (black arrows).

Four days later, the vision deteriorated dramatically alongside the development of significant ophthalmic signs. The distant vision without correction was hand motion in the right eye and counting fingers at a distance of 1 meter in the left, with impaired color vision (0/15 on the Ishihara chart). The pupils were equal, round, and reactive to light. Fundus examination revealed a newly developed bilateral central white cloudy inner retinal opacification resembling a cherry red spot as well as central retinal thickening seen on indirect fundus biomicroscopy. Central cotton wool spots and blot hemorrhages were also demonstrated (Figure 1).

**Figure 2:**
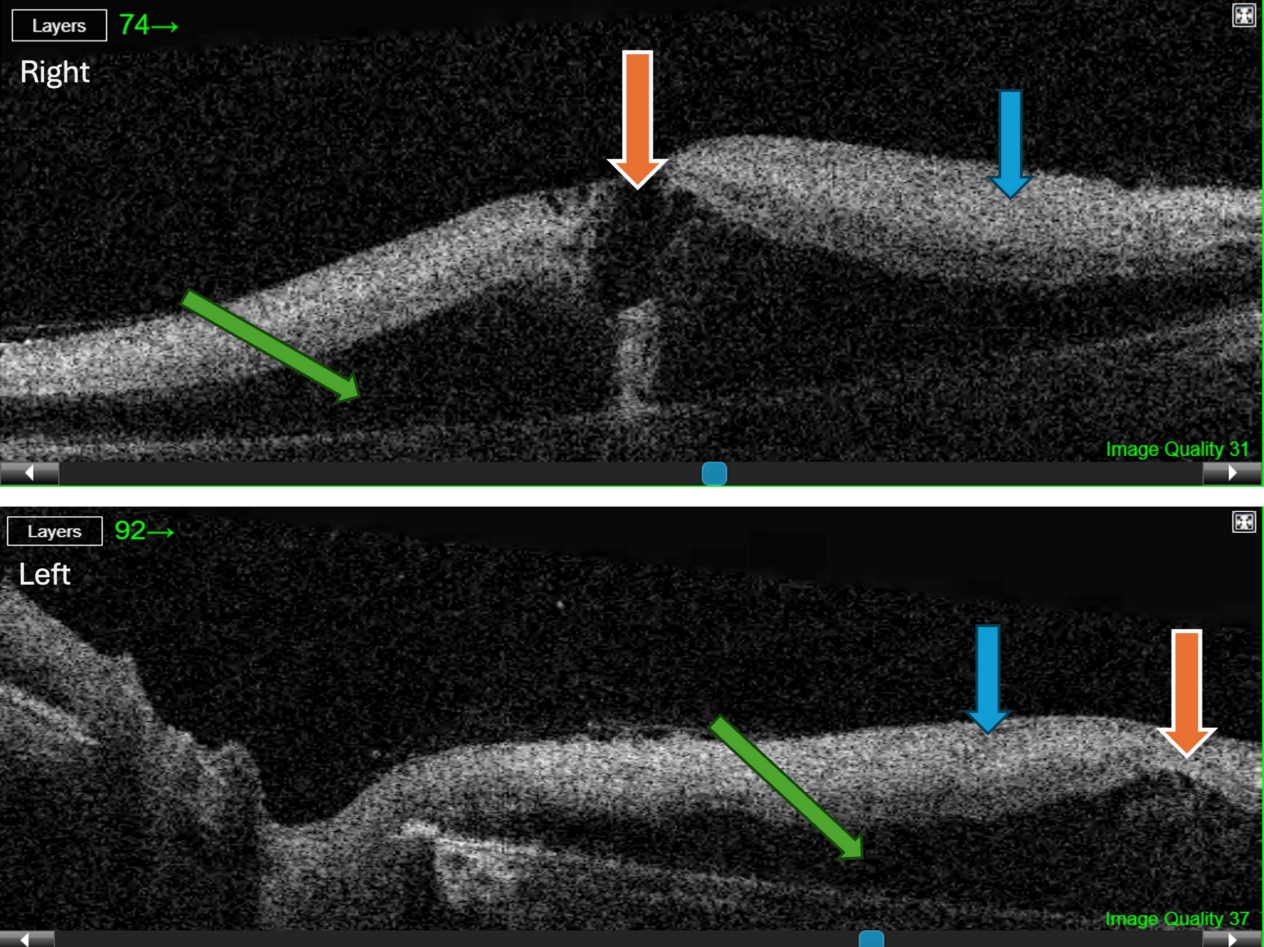
Optical coherence tomography (OCT) of the right (upper image) and left (lower image) eyes four days after symptom onset. Bilateral central inner retinal thickening and opacification with relative loss of normal inner retinal architecture is shown (blue arrow), as well as preserved foveal contour (orange arrows) and diffuse central retinal edema (green arrows).

Optical coherence tomography (OCT) revealed bilateral central inner retinal thickening and opacification with a relative loss of the normal inner retinal architecture (Figure 2).

Although the foveal contour was preserved, diffuse central retinal edema was found. These findings suggest a combined branch retinal artery and vein occlusion, specifically ciliomacular artery and vein occlusion, based on the central distribution of the findings.

**Figure 3:**
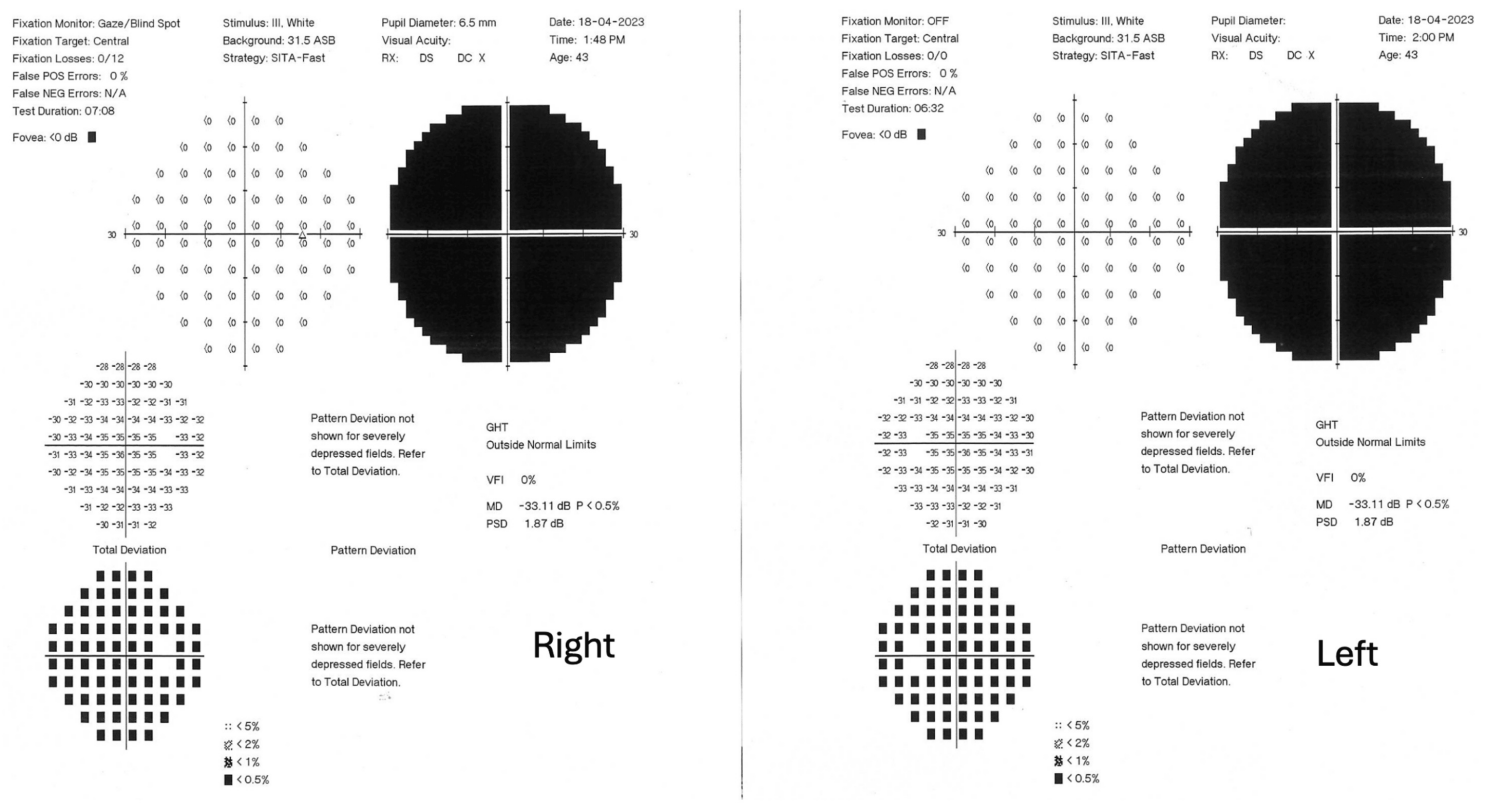
Static perimetry Humphrey Field report of 30-2 threshold protocol. This shows severe bilateral diminution of generalized retinal sensitivity and absolute central scotoma with high probability.

To exclude subclinical anterior ischemic optic neuropathy associated with tacrolimus, static automated perimetry examination was performed via Humphrey automated perimetry with a 30-2 central examination protocol. Despite the questionable reliability of the test due to low vision, it showed severe bilateral diminution of generalized retinal sensitivity and absolute central scotoma with a high probability (Figure 3).

**Figure 4:**
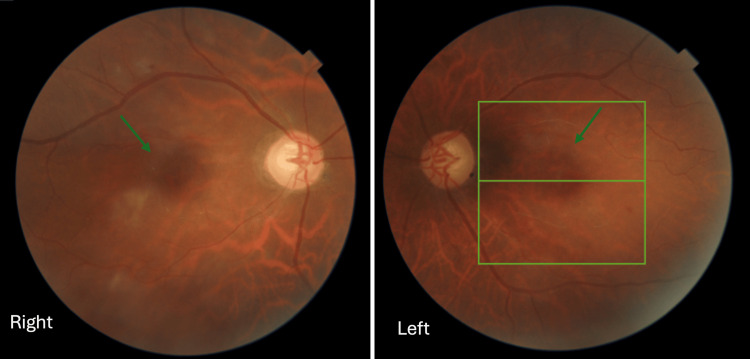
Color fundus photography two months after symptom onset. Resolution of the central retinal cloudiness is seen (green arrows).

Tacrolimus-induced ophthalmic toxicity was suspected. Thus, tacrolimus was discontinued, and ciclosporin was started. Both prednisolone and mycophenolate were continued. Over the next few weeks, the patient noticed a gradual, slight subjective improvement. On follow-up after two months, there was a partial improvement in visual acuity to counting fingers at 3 meters in both eyes. Repeat fundus examination showed total disappearance of retinal signs described earlier (Figure 4).

**Figure 5:**
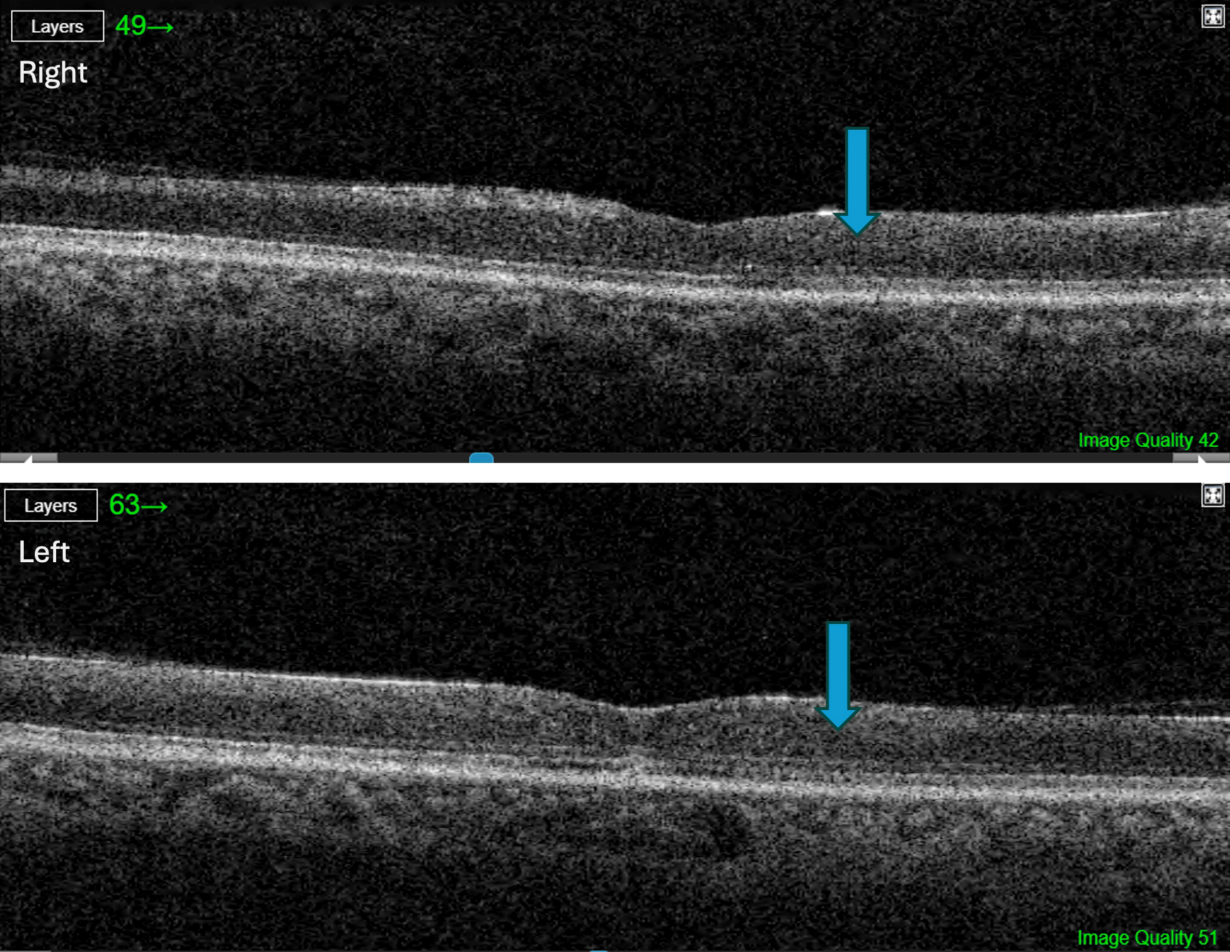
Follow-up optical coherence tomography (OCT) of the right (upper image) and left (lower image) eyes after two months. There are evident signs of bilateral ischemic maculopathy with a very thin central retina and loss of normal inner retinal architecture (blue arrows).

OCT showed signs of bilateral ischemic maculopathy with a very thin central retina and loss of the normal inner retinal architecture (Figure 5).

**Figure 6:**
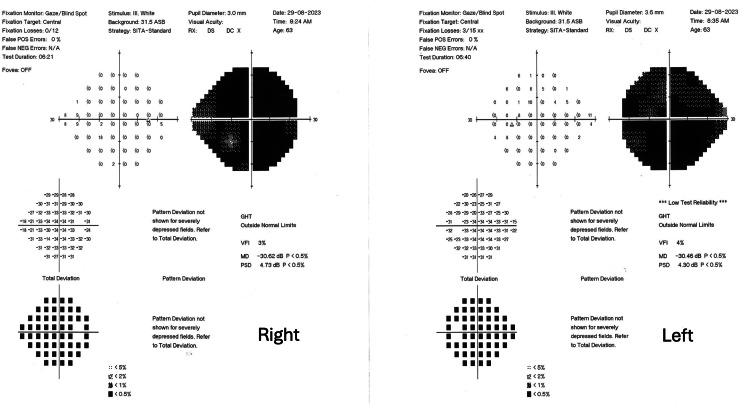
Follow-up perimetry after four months. There is significant improvement of the central scotoma with an area of relativity of moderate probability.

Perimetry showed a significant improvement in central scotoma showing an area of relativity of moderate probability (Figure 6).

## Discussion

The first case of tacrolimus-associated vision loss was reported in 1993, wherein a patient developed bilateral painless vision loss eight days after liver transplantation [3], with white matter lesions seen on MRI. These resolved after tacrolimus was discontinued, in keeping with a diagnosis of PRES. In 2000, tacrolimus was also found to cause vision loss due to optic neuropathy [7]. Thereafter, multiple cases of tacrolimus-induced optic neuropathy (TION) have been reported [8-10]. These cases demonstrated signs of direct optic nerve involvement, such as poor pupillary reaction to light, optic disc edema, pallor, or atrophy.

Tacrolimus-related maculopathy was first reported in 2011 [4] in a 63-year-old male with bilateral blurring of vision two years after liver transplantation. He had severely reduced visual acuity (20/200) in both eyes, alongside central scotoma on visual field testing. Tacrolimus was discontinued; however, there was no information on follow-up. Similarly, Santarelli et al. described a case with unilateral (left eye) visual disturbance but with a less severe presentation in a patient taking extended-release tacrolimus 10 months after liver transplantation [5]. The tacrolimus level was normal at the time of presentation. Workup revealed a left central scotoma on visual field testing and thinning of the outer nuclear retinal layer on OCT. Tacrolimus was replaced with ciclosporin and mycophenolate mofetil. On follow-up, the visual disturbance and left central scotoma resolved, but the anatomical changes on OCT persisted.

Two theories exist by which tacrolimus can cause neurotoxicity and optic neuropathy. The first theory involves direct neurotoxicity to oligodendrocytes, leading to demyelination. This was proven by Venneti et al., who demonstrated biopsy evidence of optic nerve demyelination in a patient with TION [11]. The second theory is that CNIs can lead to excess endothelin, in turn causing vasoconstriction and tissue ischemia [12]. This theory is supported by cases of TION with evidence of ischemia on fluorescein angiography [10,13]. We postulate that the ischemic maculopathy in our case is attributed to vasoconstriction in a ciliomacular artery through a similar mechanism.

There is no specific timing for TION, and in most reported cases, the patients’ tacrolimus levels were within the target range [14]. Our patient had a normal level of tacrolimus at the time of presentation, but 10 days prior, the level was higher (22 ng/mL), needing dose adjustment. Similarly, in the case reported by Santarelli et al., the patient had high serum tacrolimus levels on routine checkups near the time that the visual symptoms started. Thus, ocular toxicity can occur at any tacrolimus level, but high levels can carry a risk of tacrolimus toxicity even after level correction.

The diagnosis of tacrolimus-associated maculopathy is difficult, and other differential diagnoses of vision loss such as metabolic, inflammatory, and infectious causes should be excluded. Our patient had a normal fundus upon initial assessment by an ophthalmologist, making it challenging to arrive at a diagnosis. Four days later, macular changes appeared on follow-up, thus prompting further assessment with OCT. Fundus fluorescein angiography can also provide important data to confirm the diagnosis, but the worsening kidney function in our patient means that contrast is relatively contraindicated. This should be avoided, especially if the diagnosis can be ascertained with clinical examination and less invasive modalities. Nevertheless, our diagnosis is strongly supported by the improvement in vision after the discontinuation of tacrolimus.

## Conclusions

This is the first case of bilateral severe early-onset tacrolimus-associated ischemic maculopathy with a subacute presentation. This disease can be explained at least partially by the occurrence of a vasoocclusive accident at the arteriolar and venular level of the ciliomacular vascular system, rather than the involvement of major vascular or capillary elements.

Despite the rarity of tacrolimus-associated vision loss, we recommend that any patient presenting with visual disturbances after kidney transplantation should undergo immediate ophthalmological assessment, and medication-related adverse effects should be considered as a differential diagnosis. Repeated fundus assessment is also recommended since the appearance of abnormal fundoscopy findings may be delayed.
